# Loss of RTN3 phenocopies chronic kidney disease and results in activation of the IGF2-JAK2 pathway in proximal tubular epithelial cells

**DOI:** 10.1038/s12276-022-00763-7

**Published:** 2022-05-20

**Authors:** Liang-Liang Fan, Ran Du, Ji-Shi Liu, Jie-Yuan Jin, Chen-Yu Wang, Yi Dong, Wan-Xia He, Ri-Qiang Yan, Rong Xiang

**Affiliations:** 1 Department of Nephrology, Third Xiangya Hospital of Central South University, Changsha, 410013 China; 2 Department of Cell Biology, School of Life Sciences, Central South University, Changsha, 410013 China; 3 Hunan Key Laboratory of Animal Models for Human Diseases, School of Life Sciences, Central South University, Changsha, 410013 China; 4 Hunan Key Laboratory of Organ Fibrosis, Third Xiangya Hospital of Central South University, Changsha, 410013 China; 5 Department of Neuroscience, University of Connecticut Health, Farmington, CT 06032 United States

**Keywords:** Chronic kidney disease, Experimental models of disease

## Abstract

Reticulon 3 (RTN3) is an endoplasmic reticulum protein that has previously been shown to play roles in neurodegenerative diseases, but little is known about its function in the kidneys. The aim of the present study was to clarify the roles of RTN3 in chronic kidney disease (CKD) and kidney fibrosis. In this study, RTN3 levels were measured in kidney tissues from healthy controls and CKD or kidney fibrosis patients. An RTN3-null mouse model was generated to explore the pathophysiological roles of RTN3 in the kidneys. The underlying mechanisms were studied in primary proximal tubular epithelial cells and HEK293 cells in vitro. The results showed that (1) a reduction in RTN3 in mice induces CKD and kidney fibrosis; (2) decreased RTN3 expression is found in patients with CKD; (3) RTN3 plays critical roles in regulating collagen biosynthesis and mitochondrial function; and (4) mechanistically, RTN3 regulates these phenotypes by interacting with GC-Rich Promoter Binding Protein 1 (GPBP1), which activates the IGF2-JAK2-STAT3 pathway. Our study indicates that RTN3 might play crucial roles in CKD and kidney fibrosis and that a reduction in RTN3 in the kidneys might be a risk factor for CKD and kidney fibrosis.

## Kidney disease: A missing protein leads to scarring

A protein (RTN3) known to be involved in neurodegenerative diseases may play a causative role in kidney fibrosis or scarring, and chronic kidney disease (CKD). An estimated 20% of CKD cases may have genetic causes and identifying the genes involved may help find better treatments. Ri-Qiang Yan at the University of Connecticut Health, Farmington, USA, and Rong Xian at Central South University, China, noticed that mice in which the gene coding for RTN3 was inactivated had kidney fibrosis. The researchers showed that RTN3 levels were also lower in kidney tissues of patients with CKD than in healthy individuals and that RTN3 levels were inversely proportional to disease progression. Further investigation showed that decreased RTN3 caused extra collagen deposition and misshapen mitochondria, the cellular powerhouses, in the kidney. These results identify a potential novel risk factor for CKD.

## Introduction

Chronic kidney disease (CKD) is a type of kidney disease in which there is gradual loss of kidney function over a period of months or years^1^. It affects 10–15% of the population worldwide and is now recognized as the most rapidly increasing contributor to the global burden of disease. The costs related to CKD and end-stage renal disease (the terminal manifestation of CKD) are an enormous burden for all healthcare systems around the world^2^. Usually, CKD does not cause symptoms until it reaches an advanced stage. At the middle and late stages of CKD, edema, fatigue, vomiting, loss of appetite, or confusion may develop^3^. In the clinic, the recommended diagnosis and testing procedures for CKD include blood pressure measurement, urine testing, and serum creatinine measurement^2^. Although many early-onset CKD cases were not previously considered to be of genetic origin, studies in recent years have discovered that approximately 20% of CKD cases may be associated with genetic factors^4^. Thus far, more than 200 candidate genes, including collagen-related genes, mitochondria-related genes, and ion channel-related genes, have been identified for 70% of CKD cases associated with genetic factors^4^.

The reticulon (RTN) protein family consists of RTN1 through RTN4 in mammals, and its members have a signature C-terminal RTN homolog domain (RHD)^5^. Biochemically, RTNs can shape the structure of the tubular endoplasmic reticulum (ER) due to the ω- (wedge-shaped) membrane topology in the N- and C-terminal domains^6^. Functionally, RTNs have been found to regulate neurite outgrowth and negatively modulate the activity of Alzheimer’s β-secretase, and they are pathologically linked to axonopathy in hereditary spastic paraplegias^7–9^. Interestingly, the functions of RTNs in human peripheral organs are still not clear. Previously, we found that increased RTN3 can lead to obesity and hypertriglyceridemia via interaction of RTN3 with heat shock protein family A (Hsp70) member 5^10^. However, it has not been established whether RTN3 expression has any effects on kidney disease.

In this study, by employing hematoxylin and eosin (HE) staining, periodic acid-Schiff (PAS) staining, picrosirius red staining, and Masson staining, we noted kidney fibrosis, including glomerulosclerosis and tubulointerstitial fibrosis, in RTN3-knockout (RTN3-null) mice at 13 months of age. Serum and urine analyses revealed that the levels of creatinine (CR) and urea nitrogen (BUN) in serum and microalbumin (mALB) in urine were overtly increased in RTN3-null mice compared to WT mice at 13 months. We then collected 29 kidney samples from different stages of CKD and four kidney biopsy samples from healthy people (kidney contusion patients). Immunohistochemistry (IHC) found that ~37.5% of CKD patients showed low expression of RTN3 in the kidneys. Combined with PAS and HE staining, these analyses revealed that the expression of RTN3 was inversely proportional to CKD progression. Additional functional studies showed that both the glomerular basement membrane and mitochondria were misshaped in the kidneys of RTN3-null mice. Reducing the expression of RTN3 activated the IGF2-JAK2-STAT3 pathway by altering the localization of GC-rich promoter binding protein 1 (GPBP1), increasing the expression of collagen and disrupting the structure and function of mitochondria, which ultimately resulted in CKD and kidney fibrosis. Hence, our study indicates that RTN3 may play a crucial role in CKD and kidney fibrosis and that a reduction in RTN3 in the kidneys may be a risk factor for CKD and kidney fibrosis.

## Materials and methods

### Mouse strains, cell lines, human tissue samples, and key reagents

RTN3-null mice were generated, and genotyping was performed as described previously^10,11^. C57BL/6J mice were purchased from the Chinese Academy of Sciences (Shanghai, China) and bred in the Department of Zoology, Central South University. Unilateral ureteral obstruction (UUO) was induced in male mice at 7–8 weeks of age. Briefly, after anesthetization with pentobarbital sodium, the left ureter of each mouse was obstructed by two-point ligation with silk. The control mice underwent a sham operation without ligation of the left ureter. The mice were euthanized 7 days after UUO, and the kidneys were harvested for subsequent analysis.

Primary proximal tubular epithelial cells were isolated from mouse kidneys as follows: (1) the kidneys were decapsulated and bisected, and the medulla was removed; (2) the remaining cortices were finely chopped using a scalpel and digested in 1 mg/ml collagenase type-II at 37 °C for 10 min; (3) the kidney digest was passed through a series of brass sieves with progressively smaller mesh openings; (4) cells were collected from the 40 µm nylon mesh and spun at 150 × *g* for 10 min; (5) the cell pellet was resuspended in medium selective for epithelial cell growth; and (6) the cells were seeded onto 1% gelatin-coated tissue culture plates and incubated at 37 °C with 5% CO_2_.

The HEK293 cell line was purchased from the Cell Bank of the Shanghai Institutes for Biological Sciences (Shanghai, China) and maintained at 37 °C in a humidified, 5% CO_2_–controlled atmosphere in DMEM supplemented with 10% fetal bovine serum, 50 IU/mL penicillin, 50 mmol/L streptomycin, and glutamine.

The study protocol was approved by the Review Board of Central South University in China.

The studies involving human participants and animals were reviewed and approved by the Third Xiangya Hospital of Central South University Ethics Committee (Approval No. 2020-S533, date 2020.9.15). Kidney tissues were collected from puncture or surgical specimens, and healthy kidney tissues were collected from patients with kidney contusions. All patients provided written informed consent.

The RTN3 antibody was generated in the Yan laboratory. Antibodies against collagen type I (Cat No: 66761-1-Ig), collagen type III (Cat no: 22734-1-AP), GPBP1 (Cat No: 21622), and STAT3 (Cat No: 60199) were purchased from Proteintech Group, Inc. Antibodies against JAK2 (# 3230S), p-JAK2 (# 3771S), and p-STAT3 (# 9145S) were purchased from Cell Signaling Technology. Antibodies against IGF2 (sc-515805) and GAPDH (sc-47724) were purchased from Santa Cruz Biotechnology. Alexa Fluor 488 (A-11008), Alexa Fluor 568 (A-11011), DAPI (62247), a BCA Protein Assay and Analysis Kit (23227), a PureLink^®^ RNA Mini Kit (12183025), and Maxima SYBR Green/ROX qPCR Master Mix (2×) (K0221) were purchased from Thermo Fisher Scientific. Antibodies against COL4A5 (ab231957) and a creatinine assay kit (ab204537) were purchased from Abcam. A Urea Nitrogen (BUN) Colorimetric Detection Kit (EIABUN) was purchased from Novo Biotechnology Co., Ltd. A mouse mALB ELISA Kit (JL26402) was purchased from Shanghai Jianglai Industrial Limited by Share Ltd. A Hematoxylin-Eosin/HE Staining Kit (G1120), Masson’s Trichrome Stain Kit (G1340), Picrosirius Red (Direct Red 80) Stain Kit (S8060), Glycogen Periodic Acid Schiff (PAS/Hematoxylin) Stain Kit (G1281), Broad Spectrum Immunohistochemistry Kit (SP0041), ATP (BC0300), Reactive Oxygen Species (ROS) Assay Kit (CA1410), Mitochondrial Membrane Potential Assay Kit with JC-1 (M8650), Dual-Lucy Assay Kit (D0010) and Nuclear Protein Extraction Kit (R0050) were purchased from Beijing Solarbio Science & Technology Co., Ltd.

### HE staining, Masson staining, and PAS staining

Paraformaldehyde-fixed kidney tissue was embedded in paraffin and sliced into 6-μm sections. The sections were stained with an HE staining kit, a Masson staining kit, and a PAS staining kit and examined by routine light microscopy. Staining was performed according to established protocols.

### Immunohistochemistry

Immunohistochemistry experiments were performed with a broad-spectrum immunohistochemistry kit (Solarbio, Beijing, China). Kidney tissues from mice and humans were sectioned in the sagittal plane at a thickness of 10 μm with a cryostat after 4% paraformaldehyde fixation and optimal cutting temperature compound embedding.

### Coimmunoprecipitation and western blot analyses

For Western blotting, kidney tissues or cells were homogenized on ice in 1% 3-[(3-cholamidopropyl) dimethylammonio]-1-propanesulfonate extraction buffer containing complete protease inhibitors (Roche Bioscience, No. 04693159001) and 0.1 mmol/L Na_3_VO_4_ to inhibit phosphatase. The homogenates were rotated for 30 min at 4 °C to ensure extraction of membrane proteins. After centrifugation at 15,000 × *g* for 120 min, the supernatants were collected, and the protein concentrations were measured with bicinchoninic acid protein assay reagent. Nuclear proteins were isolated with a nuclear protein extraction kit according to the manufacturer’s instructions. Equal amounts of protein lysates were resolved by 4–12% Bis-Tris NuPAGE gel electrophoresis followed by standard Western blotting with the antibodies specified above. The chemiluminescence signals were scanned, and the integrated density values were calculated with a chemiluminescence imaging system (Alpha Innotech).

For coimmunoprecipitation (co-IP), the kidney tissues of WT mice were lysed, and equal amounts of lysates (500 μg in 1 mL) were used for immunoprecipitation with Protein A + G beads (P2108, Beyotime) overnight. The extensively washed immunoprecipitates were resolved by 4–12% NuPage Bis-Tris gel electrophoresis followed by standard Western blotting with the antibodies specified above. The chemiluminescence signals were scanned, and the integrated density values were calculated with a chemiluminescence imaging system (Alpha Innotech).

### Transmission electron microscopy and immunofluorescence confocal microscopy

For transmission electron microscopy (TEM), samples were prepared as previously described with modifications^11^. After dissection and fixation, each sample was sectioned to generate 70-nm-thick sections with an ultramicrotome (EM UC7; Leica Microsystems) and stained with uranyl acetate and lead citrate. Images were acquired by TEM (H-7650; Hitachi). Five samples were prepared for each experimental condition.

For confocal imaging, the cultured cells were fixed with 4% paraformaldehyde and treated with 0.5% Triton X-100. The cells were stained with relevant antibodies or using a JC-1 kit and examined under the Leica SP5 platform according to standard methods.

### ATP assay and ROS assay

Adenosine triphosphate (ATP) assays and reactive oxygen species (ROS) assays were performed using appropriate assay kits. Phosphomolybdic acid colorimetry was employed to detect the levels of ATP. The generation of ROS was determined by fluorometric analysis using 2,7-dichlorofluorescein diacetate. At least 3 independent repeats were made for each indicated cell line.

### RNA-seq and mass spectrometric analysis

Total RNA was extracted from mouse kidney tissues with a PureLink^®^ RNA Mini Kit. The Novogene Bioinformatics Institute (Beijing, China) conducted the main part of the RNA-seq and bioinformatics analysis. Real-time PCR validation was carried out in a Fast 7500 Real-Time PCR System (Applied Biosystems) using Maxima SYBR Green/ROX qPCR Master Mix (2×). Mass spectrometric analysis was also conducted at the Novogene Bioinformatics Institute (Beijing, China).

### Real-time PCR

cDNA was synthesized from a total of 1 μg of RNA using a RevertAid First Strand cDNA Synthesis Kit (Thermo Fisher Scientific, #K1621) with oligo(dT) primers. Real-time PCR was carried out in a Fast 7500 Real-Time PCR System (Applied Biosystems) using Maxima SYBR Green/ROX qPCR Master Mix (2×) (Thermo Fisher Scientific, #K0221). The 2^(−ΔΔCt)^ method was used to compare the mRNA expression between the affected individuals and the controls. Each assay was performed in five independent tests.

### Dual-luciferase reporter experiment

The CDS of GPBP1 was cloned into pcDNA3.1 by PCR, and the promoter of IGF2 was cloned into a luciferase plasmid (Luc promoter (IGF2)). Both plasmids were cotransfected into HEK293 cells according to the instructions of the Dual-Lucy Assay Kit. Luciferase activity was detected on a SpectraMax L microplate system.

### Statistical analysis

The data were subjected to statistical analysis with Graph-Pad Prism 5 (GraphPad Software) and plotted with AI Illustrator (Adobe). The results represent the mean ± SEM from at least three independent experiments, as indicated in the figure legends. Two-tailed Student’s *t* tests based on ANOVA were used for 2-group comparisons. For real-time PCR analysis, we used the ΔΔCT method. Differences were considered statistically significant at *P* < 0.05, with significance indicated in the figures as *P < 0.05, **P < 0.01, and ***P < 0.001 (ns represents no significant difference).

## Results

### RTN3-null mice exhibit a phenotype related to CKD

**Fig. 1:**
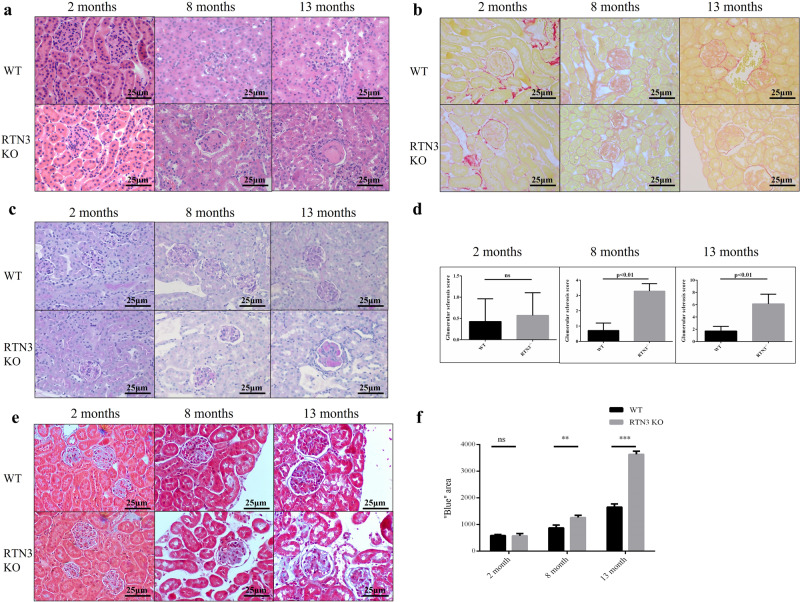
RTN3-null mice presenting phenotypes of CKD and kidney fibrosis. **a** HE, **b** picrosirius red, and **c** PAS staining analysis showing the glomeruli in WT (*n* = 5) and RTN3-null mice (*n* = 5) of different ages. **d** Statistical evaluation of glomerulosclerosis in WT and RTN3-null mice of different ages. **e**, **f** Masson staining illustrating the distribution of collagen in the kidney tissues of WT (*n* = 5) and RTN3-null mice (*n* = 5) of different ages.

RTN3-null mice were previously generated to investigate the role of RTN3 in the regulation of lipid droplet expansion and the formation of dystrophic neurites^10,11^. We have also reported a specific isoform (RTN3-C) in mouse kidneys^11^. Hence, we hypothesized that RTN3 may play a crucial role in the development of the kidneys. We collected kidneys from RTN3-null and WT mice maintained on standard chow at three different ages for histology. HE, picrosirius red and PAS staining indicated that, compared to WT mice, RTN3-null mice presented with overt glomerulosclerosis at 8 months of age and developed hyalinosis at 13 months (Fig. 1a–d). Masson staining revealed that, compared to WT mice, RTN3-null mice began to show kidney fibrosis at 8 months, which further progressed at 13 months (Fig. 1e, f). Glomerulosclerosis and kidney fibrosis showed age-dependent progression in RTN3-null mice.

To further confirm the phenotypes related to CKD in RTN3-null mice, we collected serum and urine to determine the levels of CR, blood urea nitrogen, and mALB from RTN3-null and WT mice at three different ages. No difference was detected between WT and RTN3-null mice at 2 months. However, at 8 months and 13 months, the levels of C, blood urea nitrogen, and mALB in RTN3-null mice were higher than those in WT controls, and the abnormality was more pronounced in 13-month-old mice than in 8-month-old mice (Supplementary Fig. 1a–c).

In addition, we generated a UUO model in WT and RTN3-null mice at 2 months. HE and Masson staining revealed that RTN3-null mice developed more severe glomerulosclerosis and kidney fibrosis than WT mice (Supplementary Fig. 2a–c), and the levels of CR were also higher in RTN3-null mice than in WT mice (Supplementary Fig. 2d). These observations suggest that RTN3 deficiency may lead to glomerulosclerosis and kidney fibrosis in an age-dependent manner, an obvious CKD phenotype.

### Link between low expression of RTN3 and CKD in humans

**Fig. 2:**
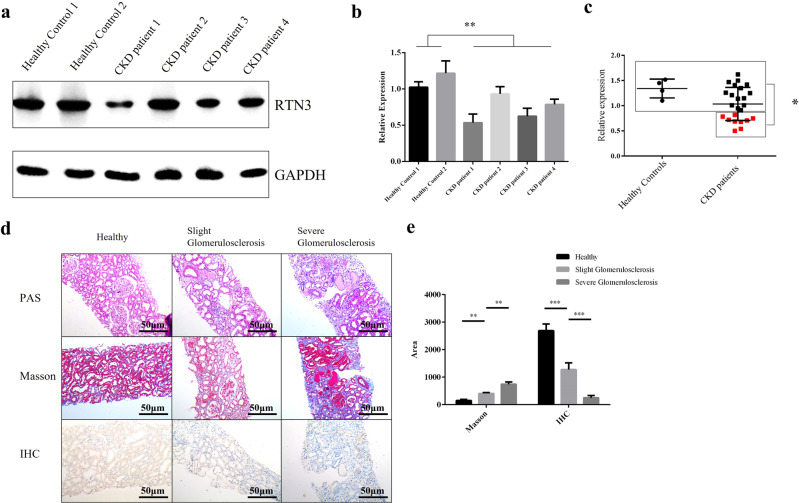
RTN3 levels are decreased in CKD patients. **a**, **b** Western blot analysis showing the expression levels of RTN3 in normal individuals (*n* = 2) and CKD patients (*n* = 4). **c** IHC analysis showing RTN3 expression levels in kidney tissues from healthy control subjects (*n* = 4) or CKD patients (*n* = 24). The red dots represent samples with lower RTN3 levels. **d** PAS, Masson, and IHC staining showing the conditions of glomerulosclerosis, kidney fibrosis, and RTN3 expression among patients with CKD of different stages. **e** Statistical analysis of Masson staining and IHC in the healthy group, slight-glomerulosclerosis group, and severe-glomerulosclerosis group.

To investigate the relationship between RTN3 and CKD in humans, we collected kidney tissues and/or biopsy samples from CKD patients (4 tissues and 24 biopsies) and three healthy controls (kidney contusion patients). Western blot analysis of kidney tissues showed that the RTN3 levels in CKD patients (four tissues) were much lower than those in healthy controls (two tissues) (Fig. 2a, b). IHC analysis further confirmed that ~37.5% (9/24) of CKD patients showed markedly lower expression of RTN3 than healthy controls, the level which was arbitrarily set as 1 for most representative cases (Fig. 2c).

We then selected three biopsy samples with low expression of RTN3 that represented different stages of CKD, as estimated by PAS and Masson staining. Combined with the IHC results, the findings revealed that the lower the expression of RTN3 was, the more advanced the CKD was (Fig. 2d, e). The findings in humans were consistent with those in mice, which further confirmed that RTN3 is an important regulator of CKD and kidney fibrosis.

### RTN3 deficiency can promote collagen synthesis and aggregation

**Fig. 3:**
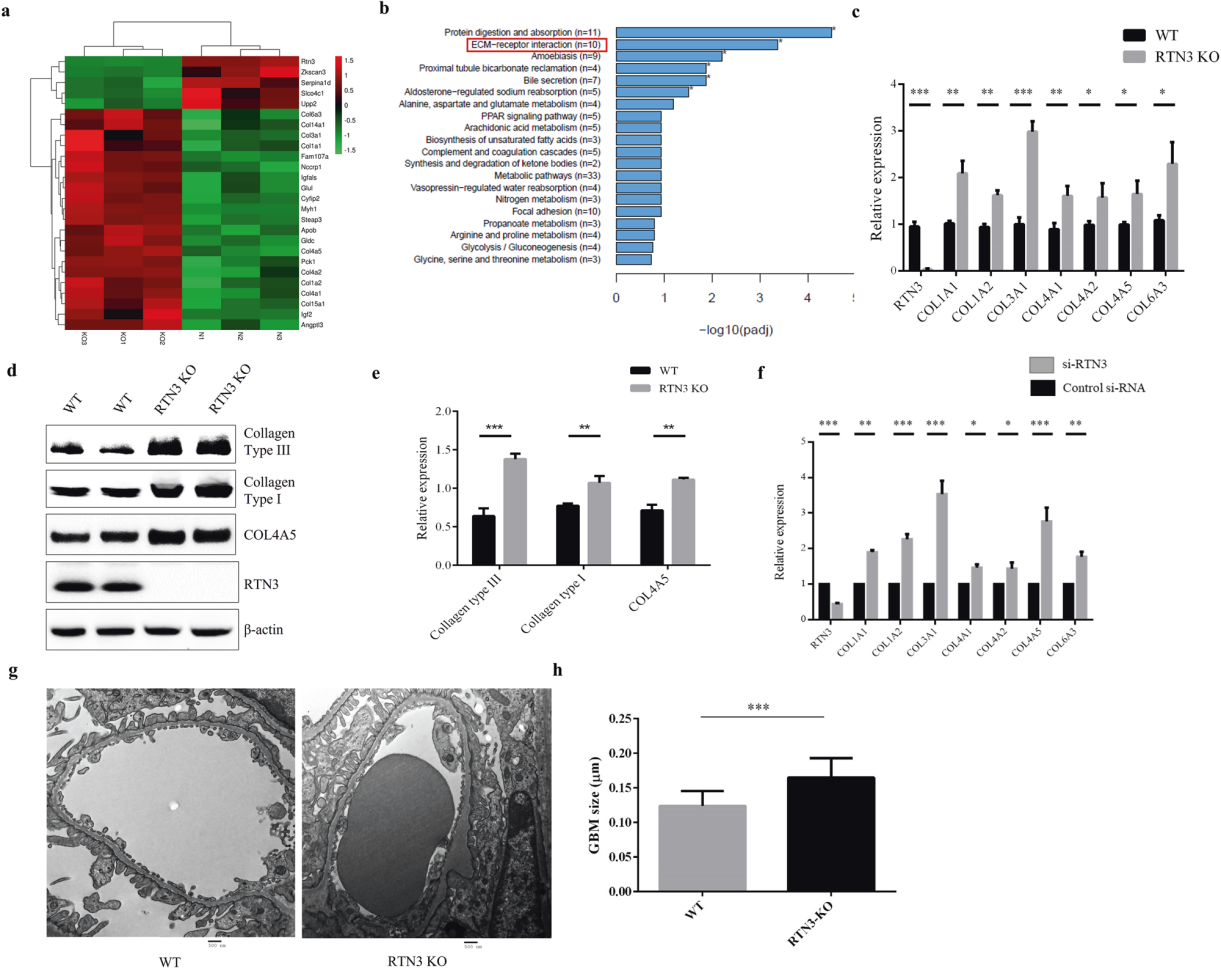
The expression of collagen is increased in RTN3-null mouse kidneys. **a** Significantly differentially expressed genes between WT and RTN3-null mouse kidneys revealed by RNA-seq data. **b** KEGG pathway enrichment analysis of differentially expressed genes. The red square indicates the ECM−receptor interaction pathway. **c** mRNA levels of collagen-related genes in WT and RTN3-null mouse kidneys at 13 months as assessed by real-time PCR. **d**, **e** Western blot analysis showing the expression of collagen type I/III, COL4A5, RTN3, and β-actin in WT and RTN3-null mouse kidneys at 13 months. **f** mRNA levels of collagen-related genes in HEK293 cells transfected with si-Control and si-RTN3. **g**, **h** TEM analysis showing the glomerular basement membranes of WT and RTN3-null mice at 13 months.

Masson staining of RTN3-null mice revealed collagen aggregation in the glomerulus and tubulointerstitium. We then prepared the renal cortices of 13-month-old RTN3-null mice and WT mice for whole-mRNA sequencing. The results suggested that the expression of collagen type I, collagen type III, and collagen type IV was increased dramatically in RTN3-null mice (Fig. 3a). KEGG pathway analysis indicated that the extracellular matrix (ECM)−receptor interaction pathway was activated (Fig. 3b). Real-time PCR and Western blot analysis further confirmed the increased expression of collagen type I, collagen type III, and COL4A5 in RTN3-null mice (Fig. 3c–e). In addition, when the expression of RTN3 was knocked down in HEK293 cells by siRNA, the expression of collagen-related genes showed changes consistent with the RNA-seq data in RTN3-null mice (Fig. 3f). In addition, we compared the glomerular basement membranes of WT and RTN3-null mice at 13 months by TEM and found that the glomerular basement membranes of RTN3-null mice presented with irregular thickening, while those of WT mice were smooth and even in thickness (Fig. 3g, h). Previous studies have suggested that increased collagen levels may disrupt the structure of the glomerular basement membrane. The data relating to collagen suggested that RTN3 deficiency might promote the synthesis and aggregation of collagen in the glomerulus and tubulointerstitium, which might ultimately lead to CKD and kidney fibrosis.

### RTN3 deficiency can disrupt the morphology and function of mitochondria

**Fig. 4:**
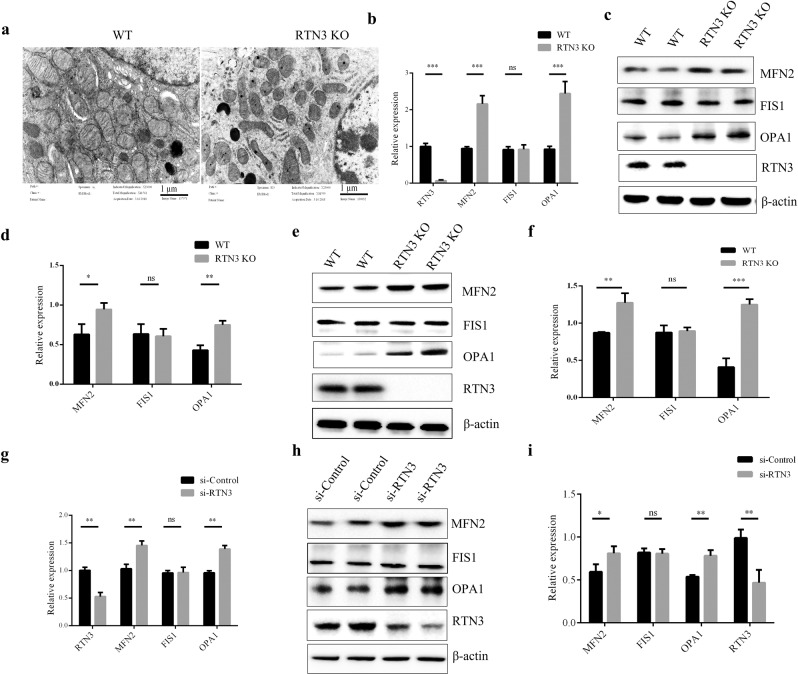
RTN3-null mouse kidneys showing mitochondrial structure damage. **a** TEM analysis showing the mitochondrial structures in renal tubular cells of WT and RTN3-null mice. **b** Real-time PCR results showing the mRNA levels of MFN2, FIS1, OPA1, and RTN3 in WT and RTN3-null mouse kidneys. Western blot analysis showing the protein levels of MFN2, FIS1, OPA1, RTN3 and β-actin in **c**, **d** WT and RTN3-null mouse kidneys and **e**, **f** WT and RTN3-null mouse primary cultured renal tubular epithelial cells. **g** mRNA levels and **h**, **i** protein levels of MFN2, FIS1, OPA1, RTN3, and β-actin in HEK293 cells transfected with si-Control and si-RTN3.

When we examined the glomerular basement membrane by TEM, we also found that the morphology of mitochondria was disrupted in the renal tubular cells of RTN3-null mice (Fig. 4a). Therefore, we examined whether there are any changes in proteins that control mitochondrial morphology. Mitofusin-2 (MFN2) and fission-1 (FIS1) are two proteins that dynamically regulate mitochondrial fusion and fission, respectively, while optic atrophy 1 (OPA1) functions to maintain the normal morphology and function of the cristae structure of the inner mitochondrial membrane^12,13^. Real-time PCR and Western blot analysis revealed that the expression of MFN2 and OPA1 was increased in RTN3-null mice compared to WT mice, but the level of FIS1 was not changed (Fig. 4b–d). We then performed Western blot analysis of renal tubular epithelial cell primary cultures isolated from RTN3-null mice and WT mice and found increased expression of MFN2 and OPA1 in RTN3-null cells (Fig. 4e, f). Knocking down RTN3 in HEK293 cells also revealed similar results at the RNA and protein levels (Fig. 4g–i). These observations demonstrated that RTN3 deficiency might affect mitochondrial morphology in renal tubular epithelial cells.

We also measured the ATP levels and ROS levels in primary cultured renal tubular epithelial cells from RTN3-null and WT mice as well as HEK293 cells transfected with si-Control and si-RTN3. The ATP levels in RTN3-null or si-RTN3 cells were noticeably lower than those in WT or si-Control cells (Supplementary Fig. 3a, b). The ROS levels were markedly increased in RTN3-null or si-RTN3 cells compared with WT or si-Control cells (Supplementary Fig. 3c, d). JC-1 staining of primary cultured cells also indicated that the mitochondrial membrane potential of RTN3-null cells was disrupted (Supplementary Fig. 3e). All these data proved that RTN3 deficiency may disrupt the function of mitochondria in renal tubular epithelial cells.

### RTN3 can regulate the IGF2-JAK2-STAT3 pathway by interacting with GPBP1 to affect collagen and mitochondria

**Fig. 5:**
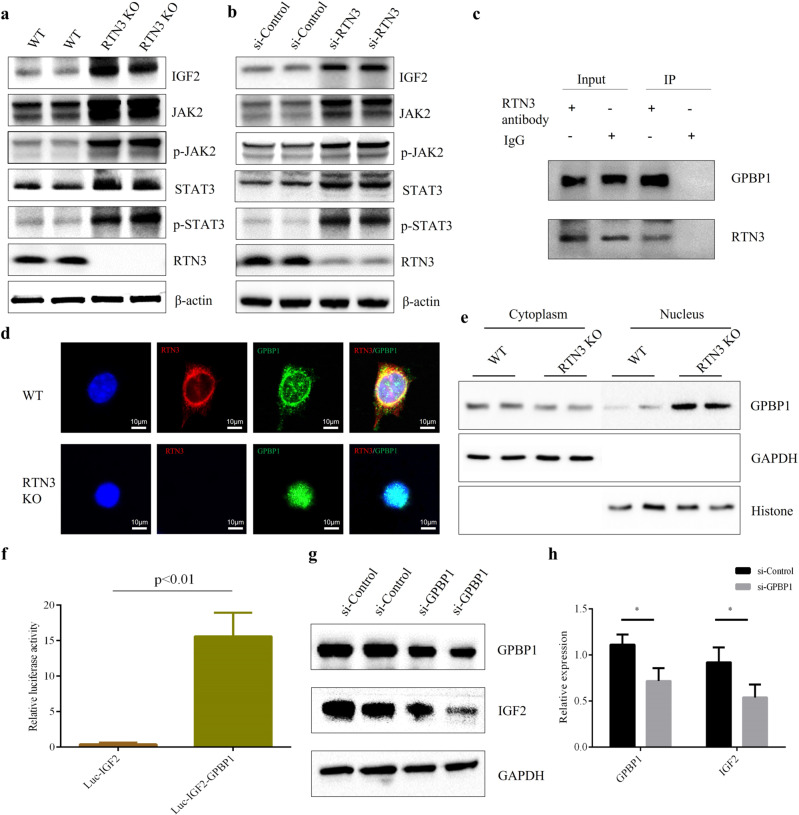
RTN3 can regulate the IGF2-JAK2-STAT3 pathway by interacting with GPBP1. Western blot analysis showing the levels of IGF2, JAK2, p-JAK2, STAT3, p-STAT3, RTN3, and β-actin in **a** WT and RTN3-null mouse kidneys and **b** HEK293 cells in the si-Control and si-RTN3 groups. **c** Coimmunoprecipitation (Co-IP) confirmed the interaction between RTN3 and GPBP1 in mouse renal tubular epithelial cells. **d** Immunofluorescence staining showing the subcellular localization of RTN3 and GPBP1 in WT and RTN3-null mouse primary cultured renal tubular epithelial cells. **e** Western blot analysis of the levels of GPBP1, GAPDH, and histone in the cytoplasm and nuclei of WT and RTN3-null mouse primary cultured renal tubular epithelial cells. **f** In vitro dual-luciferase reporter assay showing the protein interaction. **g**, **h** Western blot analysis showing the levels of GPBP1 and IGF2 in RTN3-null cells.

We confirmed that RTN3 deficiency might induce CKD and kidney fibrosis by affecting collagen and mitochondria. However, it was not clear how RTN3 regulated collagen synthesis and aggregation and mitochondrial morphology. We reanalyzed the RNA-seq data and found that the expression of insulin-like growth factor 2 (IGF2) was dramatically increased in the kidneys of RTN3-null mice compared to WT mice (Fig. 3a). IGF2 is an important protein in kidney development^14^. Upregulation of IGF2 in nephron progenitor cells may lead to Perlman syndrome, a rare disease including serious kidney disease^15^. Signals downstream of IGF2 are mediated by the JAK-STAT pathway^16^. Real-time PCR and Western blot analysis revealed that the expression of IGF2, JAK2, p-JAK2, STAT3, and p-STAT3 was increased in RTN3-null mouse kidneys and si-RTN3 HEK293 cells (Fig. 5a, b, Supplementary Fig. 4a–d). Activation of JAK2-STAT3 has been proven to promote collagen synthesis and aggregation^17,18^. In addition, the morphology and function of mitochondria can also be controlled by the JAK2-STAT3 pathway^19,20^. Hence, we hypothesize that RTN3 deficiency may activate the IGF2-JAK2-STAT3 pathway and affect collagen synthesis and aggregation and the morphology and function of mitochondria, which ultimately leads to CKD and kidney fibrosis.

However, how can RTN3, an ER protein, regulate the IGF2-JAK2-STAT3 pathway? We isolated total proteins from renal tubular epithelial cells of WT mice to perform co-IP and mass spectrometric analysis. We identified GPBP1 as a novel RTN3-interacting protein, which was validated by co-IP (Fig. 5c). Immunofluorescence staining showed that GPBP1 localized to both the nucleus and the cytoplasm in renal tubular epithelial cells, but in RTN3-deficient cells, GPBP1 localization was altered, and most GPBP1 was in the nucleus (Fig. 5d). Western blot analysis of the proteins isolated from the cytoplasm and the nucleus also showed that more GPBP1 protein localized in the nucleus in RTN3-null cells than in WT cells (Fig. 5e, Supplementary Fig. 4e). Previous studies have proven that GPBP1 functions as a GC-rich promoter-specific transactivating transcription factor^21^. The interaction of GPBP1 with the GC-rich promoter of IGF2 was assessed by dual-luciferase reporter assay in vitro (Fig. 5f). Knocking down GPBP1 in RTN3-null cells with siRNA also decreased the expression of IGF2, which also proved that GPBP1 was an important link between RTN3 and IGF2 (Fig. 5g, h).

**Fig. 6:**
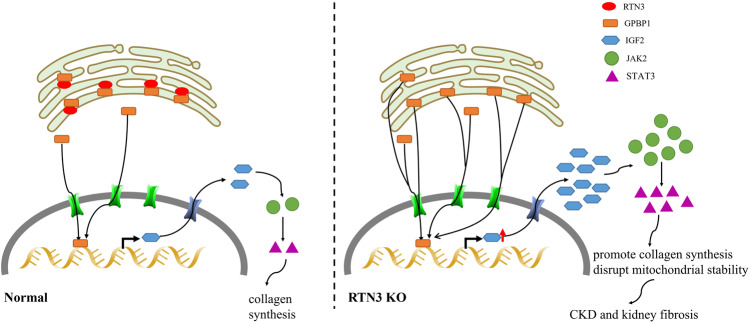
The mechanism by which RTN3 regulates the IGF2-JAK2 signaling pathway in CKD and kidney fibrosis. Potential mechanism by which decreased reticulon 3 (RTN3) expression induces CKD and kidney fibrosis.

Collectively, our data suggest that a fraction of GPBP1 can localize in the ER by interacting with RTN3 in kidney cells. When RTN3 is decreased, bound GPBP1 may be released and enter the nucleus, which may activate the transcription of IGF2. The increased IGF2 can activate the JAK2-STAT3 pathway, promote collagen synthesis and aggregation and impair mitochondria, ultimately resulting in CKD and kidney fibrosis (Fig. 6).

## Discussion

RTN3 is an ER membrane protein that exerts various biological functions; for example, it modulates Aβ levels, apoptosis, and autophagic responses^22–24^. This protein is broadly expressed and has multiple splice variants^11^. Whether this tubular ER-related protein is involved in the health and disease of the kidneys has never been reported. In this study, we obtained clinical and genetic evidence that RTN3 deficiency in kidney tissue might lead to CKD and kidney fibrosis. Our results suggest a causative relationship between RTN3 decreases and CKD in patients.

The mechanism by which RTN3 deficiency leads to CKD merits further exploration. In this study, we identified GPBP1 as a novel RTN3-interacting protein. Without RTN3, GPBP1 cannot be anchored to the endoplasmic reticulum membrane, and a fraction of GPBP1, a GC-rich promoter-specific transactivating transcription factor^21^, may enter the nucleus to activate the expression of IGF2, a protein known to be a key regulator of kidney diseases^14^. Increased IGF2 can activate the JAK2-STAT3 pathway, which has been proven to induce CKD and kidney fibrosis by regulating collagen levels and mitochondrial function^17–19^. Aggregation of collagen and disruption of mitochondria are crucial events in the pathogenesis of CKD and kidney fibrosis. In addition, some studies have revealed that GPBP1 can affect glomerular basement membrane collagen organization in the cytoplasm^21^. Without RTN3, the localization of GPBP1 may be changed from the cytoplasm to the nucleus, which may also disrupt the formation of the glomerular basement membrane and lead to CKD and kidney fibrosis.

Among the RTN family proteins, four members share a RHD with similar functions in their C-terminal domains^5^. It has been demonstrated that RTNs can form homodimers or heterodimers and activate different downstream signaling pathways. Previous studies have proven that overexpression of RTN1 induces ER stress by interacting with PERK and mediates the progression of kidney disease and kidney fibrosis^25–27^. Polymorphisms of the RTN4 3′-UTR are associated with clear cell renal cell carcinoma^28^. However, to date, an association of RTN3 with kidney disease has not been reported. Our study may be the first to identify a relationship between the RTN3 gene and kidney diseases.

Insulin-like growth factors (IGF-1 and IGF-2) are necessary for normal growth and development^14^. Previous studies have revealed that IGFs play crucial roles in cell proliferation, differentiation, and survival as well as exerting insulin-like metabolic effects in most cell types and tissues^29,30^. Disruption of IGF2 has been proven to be related to different types of kidney diseases. Genetic variation in the H19-IGF2 cluster may confer a risk of impaired renal function, and loss of function of IGF2 may lead to diabetes^31^, an important risk factor linked to diabetic kidney disease (DKD). In addition, upregulation of IGF2 has been proven to lead to Perlman syndrome, a disease related to kidney injury, in mouse nephron progenitor cells^15^. In proximal tubule cells, increased IGF levels promote fibronectin expression through a pathway involving Nox-dependent ROS generation and Akt signaling^32^. In our study, the expression of IGF2 was dramatically increased in RTN3-null mice with CKD, which indicated that kidney injury might have occurred via the IGF2 pathway.

JAK2-STAT3 activation has been detected in several types of kidney disease, such as acute kidney injury, CKD and DKD^33–35^. Podocyte-specific JAK2 overexpression has been found to accelerate disease progression in a DKD mouse model^36^, and a STAT3 inhibitor (S3I-201) can attenuate fibrosis and inflammation in UUO kidneys^37^. In a CKD mouse model, activation of the JAK2-STAT3 pathway has been found to induce oxidative stress and aggravate CKD^38^. In acute kidney injury and DKD, activation of the JAK2-STAT3 pathway also promotes the immune inflammatory response and induces apoptosis^33^. Furthermore, administration of JAK2 inhibitors, including baricitinib and Huang Gan formula, effectively improves kidney function in CKD and DKD, which indicates that JAK2 inhibitors might be useful as new therapies for CKD and DKD^38,39^.

Oxidative stress is harmful to cells due to excessive generation of ROS, which has been proven to lead to CKD^40^. Disruption of mitochondrial structure and function may promote the oxidative stress response^41,42^. In our study, we detected disruption of mitochondria in RTN3-null mouse kidneys. Previous studies have revealed that activation of the JAK2-STAT3 pathway may induce mitochondrial dysfunction and oxidative stress by disrupting the balance between BCL2 and BAX^43,44^. A specific JAK2 inhibitor can attenuate TNF-α-induced oxidative stress in renal tubular epithelial cells^45^. Hence, the mitochondrial dysfunction in RTN3-null mouse kidneys described in our current study may also have occurred via the JAK2-STAT3 pathway.

In summary, our study suggests that the RTN3-null mouse model might be an ideal model for research on CKD and kidney fibrosis because the model recapitulates features of CKD. Reduced RTN3 expression is a potential risk factor for glomerulosclerosis and tubulointerstitium in the kidney because it promotes collagen synthesis and aggregation and impairs mitochondrial structure and function, partly through alteration of the localization of GPBP1 and activation of the IGF2-JAK2-STAT3 pathway. Hence, our findings shed light on the importance of the relationship between ER proteins and CKD/kidney fibrosis in humans and animals. Collectively, our data suggest that RTN3 is a key molecule in the kidneys.

## Supplementary information

Supplemental Materials

## Data Availability

The data that support the findings of this study are available from the corresponding author upon reasonable request.
