# Supplementary material for: Loss of RTN3 phenocopies chronic kidney disease and results in activation of the IGF2-JAK2 pathway in proximal tubular epithelial cells
Source: Exp Mol Med. 2022 May 20;54(5):653–61. doi: 10.1038/s12276-022-00763-7 (PMC9166791; doi:10.1038/s12276-022-00763-7)
Supplement: Supplementary file 1 — Supplemental Materials [file 12276_2022_763_MOESM1_ESM.pdf]

Loss of RTN3 phenocopies chronic kidney disease and results in activation of the IGF2-JAK2 pathway in proximal tubular epithelial cells

Running title: loss of RTN3 may lead to CKD

Liang-Liang Fan[1,2,3#]; Ran Du[1,2#]; Ji-Shi Liu[1,4#]; Jie-Yuan Jin[1,2]; Chen-Yu Wang[1,2]; Yi Dong[1,2]; Wan-Xia He[5]; Ri-Qiang Yan[5*]; Rong Xiang[1,2,3,4*]

[1] Department of Nephrology, The Third Xiangya Hospital of Central South University, Changsha, 410013, China;

[2] Department of Cell Biology, The School of Life Sciences, Central South University, Changsha 410013, China;

[3] Hunan Key Laboratory of Animal Models for Human Diseases, School of Life Sciences, Central South University, Changsha, 410013, China;

[4] Hunan Key Laboratory of Organ Fibrosis, The Third Xiangya Hospital of Central South University, Changsha, 410013, China.

[5] Department of Neuroscience, University of Connecticut Health, Farmington, CT, 06032, USA;

[#] These authors contributed equally to this study.

* Correspondence:

Ri-Qiang Yan, Ph.D

Email: riyan@uchc.edu

Department of Neuroscience, University of Connecticut Health, Farmington, CT, 06032, USA;

and

Rong Xiang, Ph.D.

Email: shirlesmile@csu.edu.cn

Department of Cell Biology, The School of Life Sciences, Central South University, Changsha 410013, China.

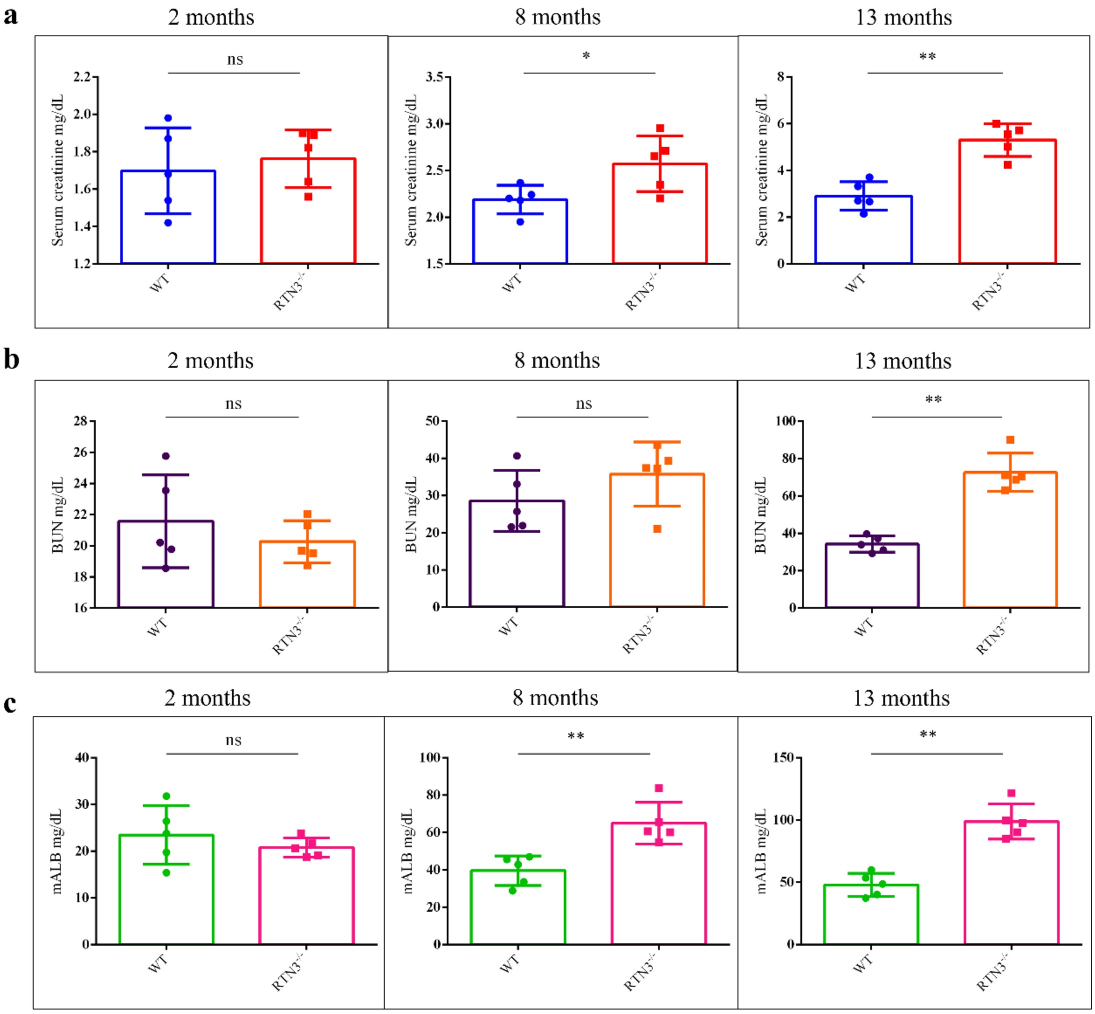

Supplementary Fig. 1. The levels of serum creatinine, blood urea nitrogen and mALB indicates RTN3-null mice are suffering from CKD. (**a**) Serum creatinine, (**b**) blood urea nitrogen and (**c**) urine mALB levels in WT (n=5) and RTN3-null mice (n=5) with different ages.

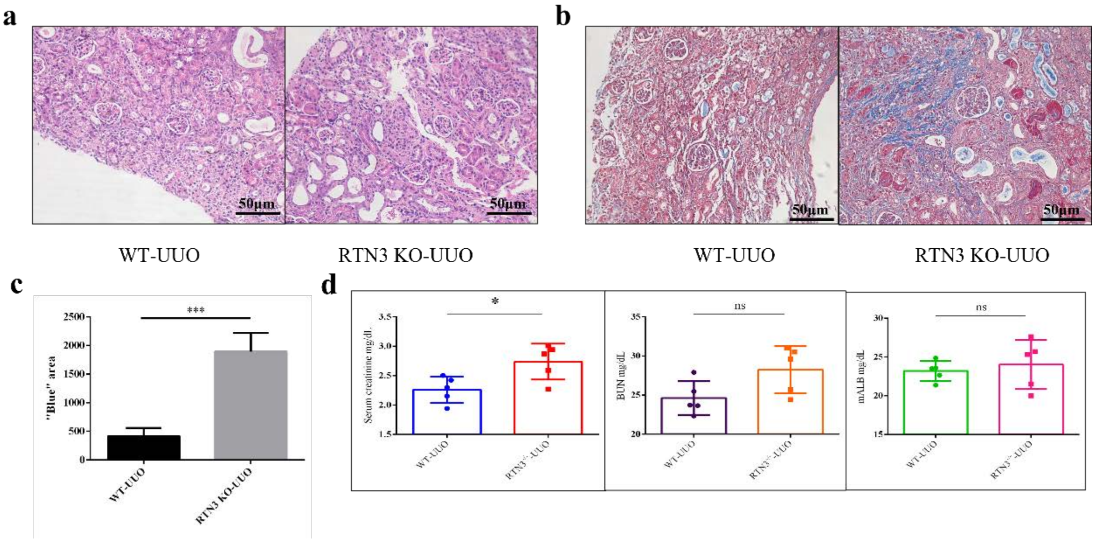

Supplementary Fig. 2. UUO models of RTN3-null mice show more severe CKD phenotypes. (**a**) H&E and (**b, c**) Masson staining exhibited the glomerular and tubular structures in WT-UUO (n=5) and RTN3-null-UUO mice (n=5) at 2 months. (**d**) Serum creatinine, blood urea nitrogen and urine mALB levels in WT-UUO (n=5) and RTN3-null-UUO mice (n=5) at 2 months.

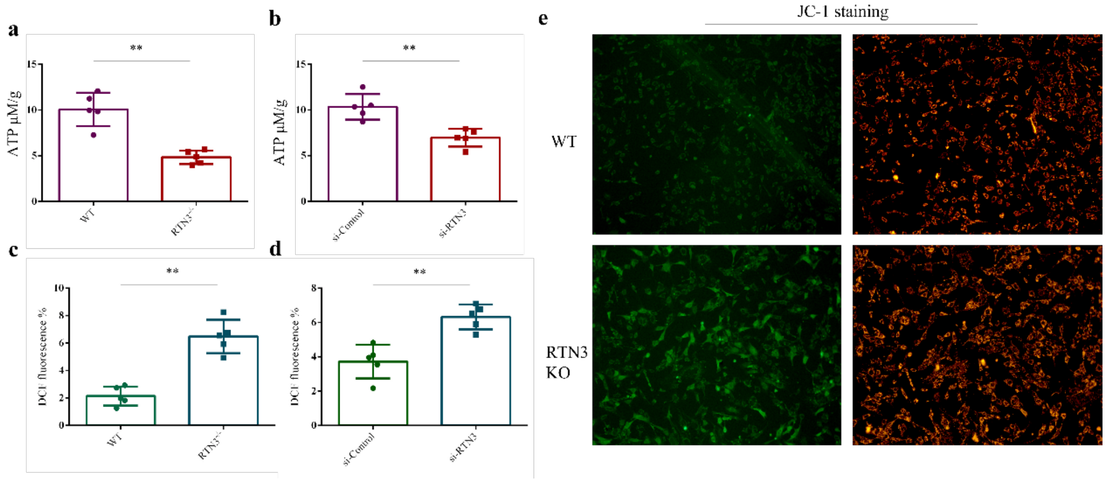

Supplementary Fig. 3. RTN3-null mice kidney shows mitochondrial dysfunction. ATP levels in (**a**) WT and RTN3-null mice primary cultured renal tubular epithelial cells group, and (**b**) HEK293 cell with si-control and si-RTN3 group. ROS levels in (**c**) WT and RTN3-null mice primary cultured renal tubular epithelial cells group, and (**d**) HEK293 cell with si-control and si-RTN3 group. (**e**) JC-1 staining in WT and RTN3-null mice primary cultured renal tubular epithelial cells.

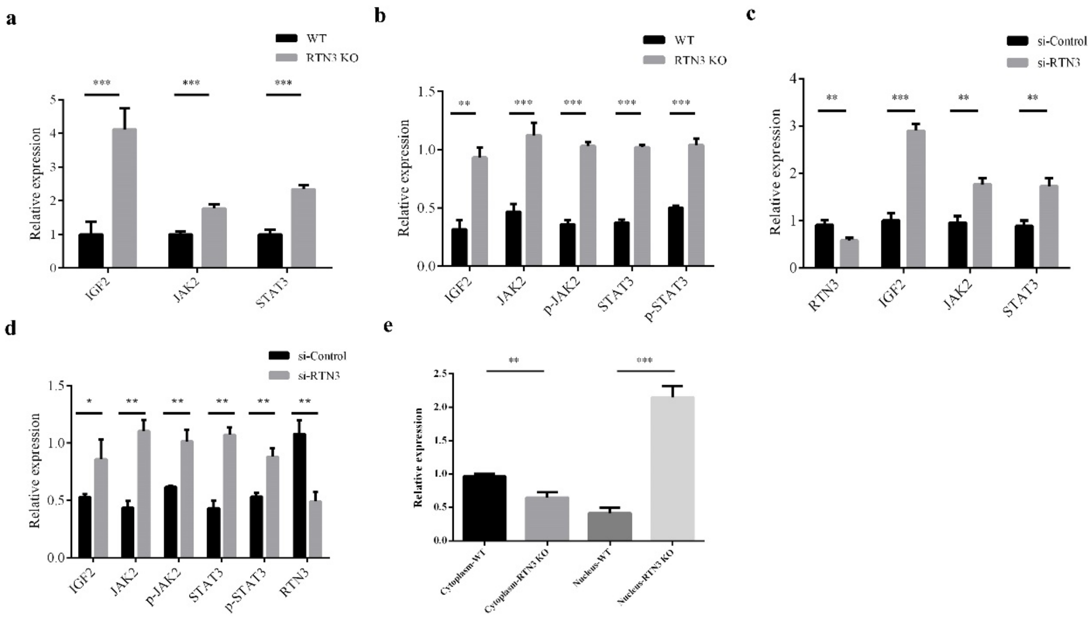

Supplementary Fig. 4. RTN3 can regulate the IGF2-JAK2-STAT3 pathway by interacting with GPBP1. (**a**) Real-time PCR analysis shows the mRNA levels of IGF2, JAK2 and STAT3 in WT and RTN3-null mice. (**b**) Statistical analysis of IGF2, JAK2, p-JAK2, STAT3 and p-STAT3 protein levels in WT and RTN3-null mice. (**c**) the mRNA levels of IGF2, JAK2 and STAT3 in HEK293 cell with si-Control and si-RTN3. (**d**) Statistical analysis of IGF2, JAK2, p-JAK2, STAT3, p-STAT3 and RTN3 protein levels in HEK293 cell with si-Control and si-RTN3. (**e**) Statistical analysis of the levels of GPBP1 in WT and RTN3-null mice primary cultured renal tubular epithelial cells cytoplasm and nucleus.
